# Injectisome T3SS subunits as potential chaperones in the extracellular export of *Pectobacterium carotovorum* subsp. *carotovorum* bacteriocins Carocin S1 and Carocin S3 secreted via flagellar T3SS

**DOI:** 10.1186/s12866-021-02405-w

**Published:** 2021-12-15

**Authors:** Huang-Pin Wu, Reymund C. Derilo, Han-Ling Chen, Tzu-Rung Li, Ruchi Briam James S. Lagitnay, Yung-Chieh Chan, Yutin Chuang, Duen-Yau Chuang

**Affiliations:** 1 Division of Pulmonary, Critical Care and Sleep Medicine, Chang Gung Memorial Hospital, Keelung, Taiwan; 2 College of Medicine, Chang Gung University, Taoyuan, Taiwan; 3 Department of Chemistry, National Chung Hsing University, 145, Xingda Rd., Taichung, 402 Taiwan; 4 College of Teacher Education, Nueva Vizcaya State University Bambang Campus, Bambang, Nueva Vizcaya Philippines; 5 College of Arts and Sciences, Nueva Vizcaya State University Bayombong Campus, Bayombong, Nueva Vizcaya Philippines; 6 Department of Entomology, National Chung Hsing University, Taichung City, Taiwan

## Abstract

*Pectobacterium carotovorum* subsp. *carotovorum* (*Pcc*) causes soft-rot disease in a wide variety of plants resulting in economic losses worldwide. It produces various types of bacteriocin to compete against related plant pathogens. Studies on how bacteriocins are extracellularly secreted are conducted to understand the mechanism of interbacterial competition. In this study, the secretion of the low-molecular-weight bacteriocins (LMWB) Carocin S1 and Carocin S3 produced by a multiple-bacteriocin producing strain of *Pcc,* 89-H-4, was investigated. Tn5 insertional mutagenesis was used to generate a mutant, TH22–6, incapable of LMWBs secretion. Sequence and homology analyses of the gene disrupted by transposon Tn5 insertion revealed that the gene *sctT*, an essential component of the injectisome type III secretion machinery (T3aSS), is required for the secretion of the bacteriocins. This result raised a question regarding the nature of the secretion mechanism of *Pcc* bacteriocins which was previously discovered to be secreted via T3bSS, a system that utilizes the bacterial flagellum for extracellular secretions. Our previous report has shown that bacteriocin Carocin S1 cannot be secreted by mutants that are defective of T3bSS-related genes such as *flhA*, *flhC*, *flhD* and *fliC*. We knocked out several genes making up the significant structural components of both T3aSS and T3bSS. The findings led us to hypothesize the potential roles of the T3aSS-related proteins, SctT, SctU and SctV, as flagellar T3SS chaperones in the secretion of *Pcc* bacteriocins. This current discovery and the findings of our previous study helped us to conceptualize a unique Type III secretion system for bacteriocin extracellular export which is a hybrid of the injectisome and flagellar secretion systems.

**Supplementary Information:**

The online version contains supplementary material available at 10.1186/s12866-021-02405-w.

## Introduction

Many bacteria often produce extracellular proteins to inhibit the growth of other competing bacterial species [1, 2]. Such extracellular proteins produced are commonly called bacteriocins. Bacteriocins are found in both Gram-positive and Gram-negative bacteria [3–5], and the functions in different bacterial species are similar. Most of the structural genes of bacteriocins (*e. g. E. coli* and *P. aeruginosa* bacteriocins) are located on the plasmid [6]. For *Pectobacterium carotovorum* subsp*. carotovorum* (*Pcc)*, the genes responsible for bacteriocins production is in the chromosomes [7–10]. When the bacterial growth environment changes and may cause harm, the structural genes in the chromosome will be activated to produce bacteriocin proteins to attack other related bacterial species. Because bacteriocin has the function of inhibiting the growth of other competing species, we can often see in some related literature that the bacteriocin protein produced by the isolated harmless bacterial species is transferred to other bacteria. Bacteriocins usually inhibit the growth of closely related harmful bacteria [11], which shows that some bacteriocins might have a high application value and economic significance.

The classification of most bacteriocins is based on the producing bacteria, molecular weight, chemical structure, and functional mechanism [3, 12, 13]. Gram-positive bacteria are often divided into four categories based on the presence of disulfide bonds or single sulfide bonds on the peptide chain of bacteriocins [14]. For the Gram-negative bacteria, however, there is no complete classification basis, so the bacteriocins are roughly classified into two, which is based on their molecular weights, i.e. high-molecular-weight bacteriocins (HMWB) and low-molecular-weight bacteriocins (LMWB). The strain *Pcc*, which is known to produce LWMBs Carocin S1 [7], Carocin D [8], Carocin S2 [9], and Carocin S3 [10], was selected in this study. These studies revealed that *Pcc* produces bacteriocins of different characteristics [7, 9]. The studies identified two significant functional proteins of the bacteriocin operon, the killing protein, which is responsible for the species’ antimicrobial activity, and the immunity protein, which is responsible for the resistance against its own antibacterial activity. The immunity protein inhibits the killer protein’s activity and protect the host cell survival. The killing proteins are organized in functional domains, with receptor-binding, translocation, and DNAse or RNAse activity [8].

When bacteria produce bacteriocins, how to deliver the killer protein outside of the cell through various secretion systems is also a topic of interest to many scholars. Several studies have shown that many Gram-negative bacteria utilize the Type III secretion system (T3SS). During the course of an infection, several Gram-negative pathogens, including *Shigella* [15], *Salmonella* [16], enteropathogenic *Escherichia coli* [17], and *Yersinia* species [18], use T3SS as injection devices to deliver multiple virulence proteins, referred to as effectors, directly into the cytosol of infected cells [19]. Moreover, findings from our previous study have shown that the secretion of the LMWB Carocin S1 from *Pcc* is dependent on the flagellar Type III secretion system which also controls the bacterium’s cell motility and cell size [20].

The T3SSs are intricate bacterial machinery that gives Gram-negative bacteria a unique and distinctive mechanism for virulence, enabling them to deliver effector proteins into their host’s cell cytoplasm bypassing the extracellular environment [21]. These protein secretion mechanisms include the injectisome (T3aSS) and flagellar (T3bSS) systems [22].

The T3bSS, being the most ancestral type of T3SS, builds the bacterial flagellum which are commonly found in both Gram-positive and Gram-negative bacteria [22, 23]. This extracellular structure enables bacterial motility in both surface and liquid environments [22, 24, 25]. An evolutionary related nanomachine of the T3SS is the T3aSS, a needle-like nanomachine that has undergone an evolution, not only for the assembly of the injectisome, but also for energizing effector proteins translocation during invasion and colonization [26–28]. The T3aSS and T3bSS share various homologies in terms of their structures. Both entail around 10 cytoplasmic and inner membrane proteins with the high resemblance in sequence and membrane topology [25–27]. These machineries have three basic structural parts: the basal body, the MS ring, and the rod that passes through the periplasmic space [26, 29–32]. These two secretion systems, however, differ in their external structure. The T3aSS features a needle with a translocon pore located at its end, while the T3bSS contains a hook and a filament [26, 31, 32]. How these two nanomachines are assembled is highly organized. They are regulated and constructed in highly explicit order. The assembly of T3aSS injectisome depends on varying affinities and kinetics of protein-protein interactions. On the other hand, assembly of the flagellum for T3bSS is based on a transcriptional hierarchy of three promoter classes [33–35].

Our previous study on the secretion mechanism of *Pcc*’s LMWB has shown that Carocin S1 is secreted via T3bSS [20]. The findings revealed that *flhA*, *flhC*, *flhD* and *fliC,* genes involved in the flagellar virulence machinery, are required for the delivery of the bacteriocin outside of the cell. However, in this current study, we found that *sctT*, a T3aSS-related gene, functions similarly in the secretion of Carocin S1 and Carocin S3 in the multiple bacteriocin-producing strain of *Pcc*. Thus, we hypothesized that the bacterium is capable of secreting bacteriocins through both the flagellar and injectosome T3SS, similar to *Burkholderia,* a bacterium capable of encoding multiple T3SSs [36, 37]. Further deletions of various genes involved in the assembly of the flagellar and injectisome T3SS, however, raised an interesting question about the intricacy in the export mechanism of these *Pcc* bacteriocins. This led us to hypothesize a unique interaction of various T3SS proteins in the secretion of *Pcc* bacteriocins.

## Results

### Isolation and detection of Tn5 insertion mutants

**Fig. 1:**
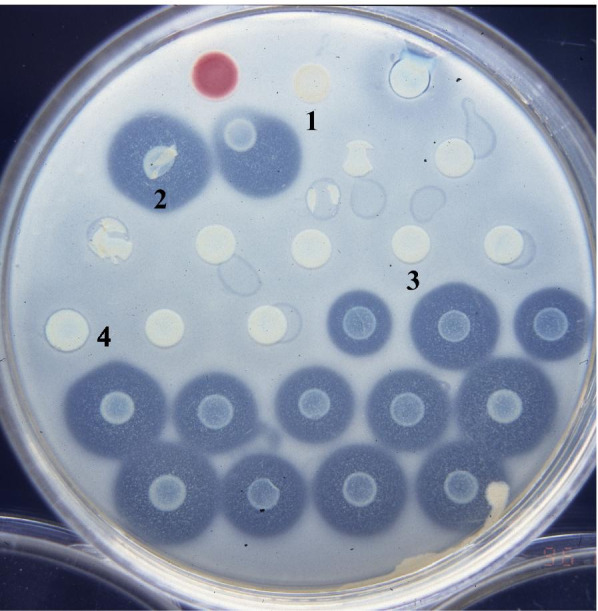
Bacteriocin activity of *P. carotovorum* subsp*. carotovorum*. The bacteriocin production of the test strains was examined by the double-layer method [38, 39]. Numbered strains: 1, *Escherichia coli* pJB4JI (containing Tn5)/1830; 2, H-rif-8-6 (wild type, parent); 3, TH22–6 (the transposon Tn5 insertional mutant); 4, TH22–10 (the transposon Tn5 insertional mutant for carocin S1) [20]. The indicator organism used in the agar overlay was *Pcc* strain Ea1068. The red colony, *Serratia marcescens*, was used as a marker, to indicate the direction and location of numbered colonies. The result showed that TH22–6 strain loses the low-molecular-weight bacteriocin activity to Ea1068

*Pcc* could undergo mutation and produce progeny strains with rifampicin resistance genes. These mutants still produce bacteriocins even after mutations. In this paper, we chose H-rif-8-6, which was from the rifampicin resistant strain 89-H-4, as the recipient of bacterial conjugation, and *E. coli* (1830) with the kanamycin resistance gene transposon Tn5 as the donor of conjugative reproduction. After mating, it is expected that the pJB4JI in *E. coli* could be transmitted into the *Pcc*. To screen the conjugated *Pcc*, we used modified Drigalski’s medium with antibiotics kanamycin and rifamycin (data not shown). Subsequently, the bacteriocin assays [38, 39] were performed to find out the strains whose low-molecular-weight bacteriocin-related genes have been successfully disrupted (Fig. 1).

The mutant strain TH22–6 has shown an indication of the Tn5 insertion. The results have shown that Transposon Tn5 has successfully disrupted the low-molecular-weight bacteriocin production-related genes in the strain [40]. Hence, we selected the mutant strain TH22–6 for further investigation. To further confirm the insertion, P3 and P4 primers were designed to amplify the specific *npt*II resistance gene on Tn5 (about 500 bp in length). In this technique, the PCR method can be used to confirm whether Transposon Tn5 has been inserted into the DNA of the wild strain (data not shown).

### Amplification of the DNA at the Tn5 insertion junction site

Two sets of primers in different directions are designed to perform Thermal Asymmetric Interlaced PCR (TAIL-PCR). This was done to produce two DNA fragments extending outward from the Transposon Tn5 gene. Since the two sets of primers are both outside the Transposon Tn5 gene, the two DNA fragments will also contain genes that may have been disrupted in addition to the Tn5 gene. The ABI PRISM DNA Sequencing System 373S (Perkin-Elmer Corporation) was used to decipher the DNA sequence.

We amplified the Tn5 insertion junction DNA from TH22–6 genomic DNA by TAIL-PCR, and one or more PCR products were obtained. The genomic DNA of *Pcc*’s insertional mutant TH22–6 was used as the template DNA. The isolated chromosomal DNA was treated with *EcoR*I and confirmed with 0.7% agarose electrophoresis gel. Using specific primers PF1 ~ 3 and PR1 ~ 3 and random primer N2 (Table 2), and the above-mentioned TH22–6 chromosomal DNA as template DNA, we performed TAIL-PCR. Supplementary Fig. 1 shows the results of the TAIL-PCR.

For TAIL-PCR products sequencing, an automated DNA sequencer 373S (ABI) was used, and a nucleotide sequence of 2249 bp was obtained. The nucleotide sequence and deduced amino acid of T3SS protein were compared by the BLAST programs of National Center for Biotechnology Information server (National Library of Medicine, USA). Sequence data were compiled with DNASIS-Mac software (Hitachi, Japan). The results show that at least two complete open reading frames (ORF1 and ORF2) were present, and Tn5 was in the ORF2, between 2248th and 2249th base pair (data not shown).

### Sequence analysis and homology

Sequence Walking was performed to identify the two DNA sequences of TAIL-PCR products. It was found that the two single-band PCR products amplified by primers PF-A3 and N2, and the single band PCR products amplified by PL3 and N1, has a total length of 2249 bp. The TAIL-PCR products amplified by specific primers PF1 ~ 3 and PR1 ~ 3 with the TAIL-PCR product amplified by specific primers PF-A1 ~ 3 and PL1 ~ 3 were also compared. Both have inserted mutations by Tn5 and the TAIL-PCR products extend outward from PCR amplification.

**Fig. 2:**
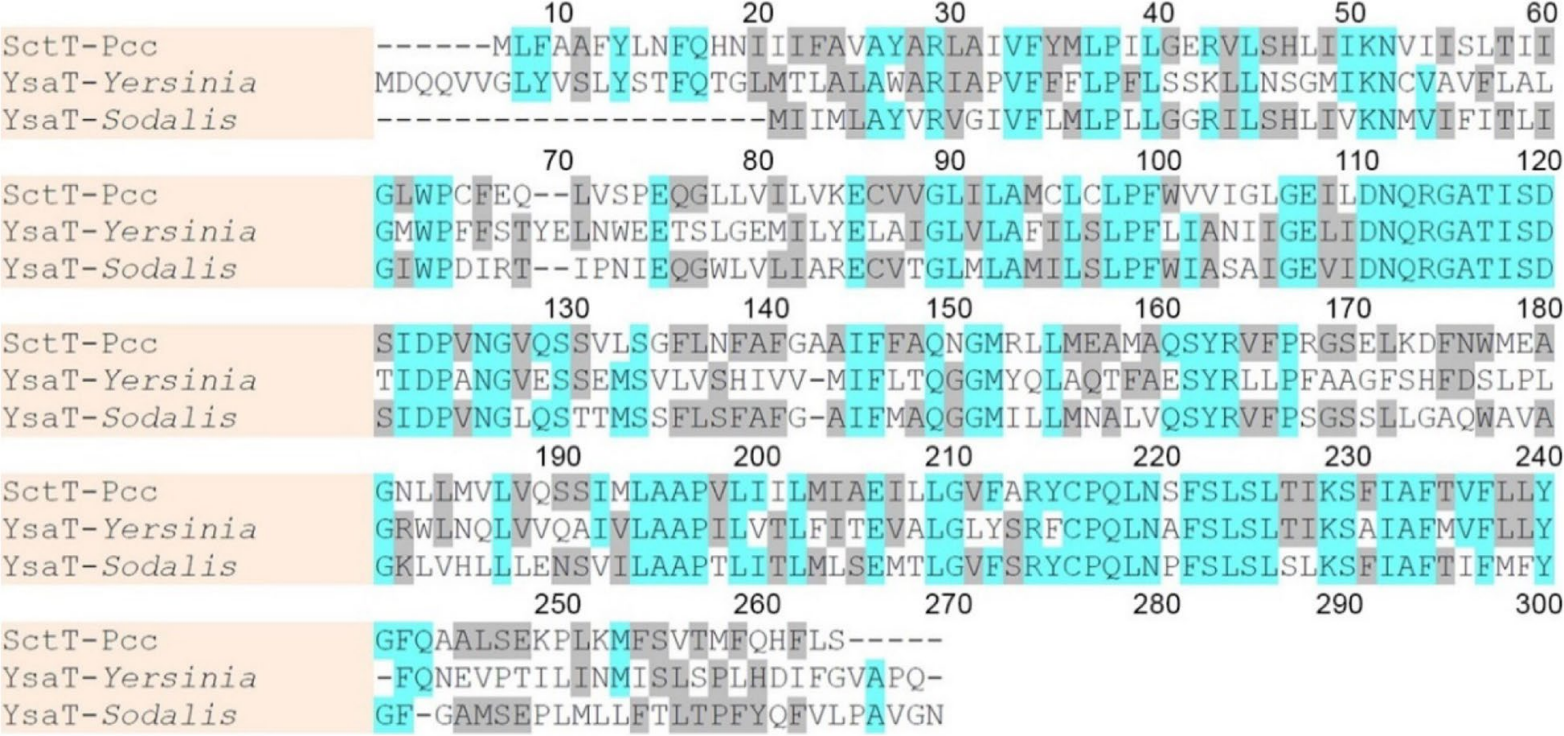
Alignment of the predicted amino acid sequence of the *Pcc sctT* gene product with the amino acid sequences of YsaT protein in *Sodalis* and *Yersinia*. Dashes are inserted to optimize the alignment

We performed sequence analysis using (BLAST) by the National Institutes of Health Medical Library (NCBI) database (Fig. 2). The result showed that ORF2 shows high homology with the YsaT protein of T3SS in *Sodalis glossinidius* (61% identity) and *Yersinia enterocolitica* (45% identity). Another high homology cassette (ORF1) in the nucleotide sequence has shown 74% identity with YsaR, also an assembly of T3SS.

**Fig. 3:**
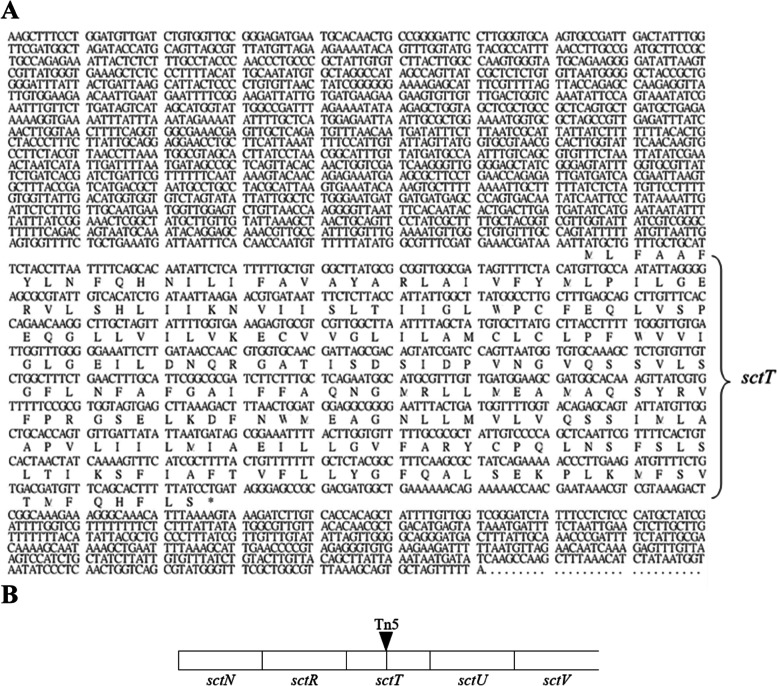
Nucleotide sequence of the *sctT* gene with the deduced amino-acid sequence. **a** The *sctT* contains 3031 base pairs; **b** The DNA sequence shows the location of the *sctT* gene, Tn5 insert and other T3aSS related genes

Based on the nucleotide sequence of TAIL-PCR product, the DNA probe (431 Probe) which was designed near the Tn5 insertion region, was hybridized with the digested wild-type strain genomic DNA (Supplementary Fig. 2). Thereafter, we sliced the DNA agarose electrophoresis gel, then purified and cloned it into pBR322 for genome library. Finally, we acquired the construction which bears the nucleotide of the interrupted gene (Supplementary Fig. 3). The nucleotide was sequenced and its length is 3031 base pairs (Fig. 3).

After compiling the sequence with DNASIS-Mac software, it indicates that ORF2 encodes 762 base pairs and a protein of 254 amino acids, which shows high homology with the YsaT protein of the T3SS in *Sodalis glossinidius* and *Yersinia enterocolitica*. The YsaT protein of these two strains is one of the essential proteins in their bacteriocin secretory systems. Hence, we speculate that the gene mutated by the insertion of Transposon Tn5 may be a gene that secretes bacteriocin. The gene was later designated as *sctT*, in accordance with the unified secretion and cellular translocation (Sct) nomenclature.

### Complementation and analysis of *flhA* and *sctT* genes

Wild-type H-rif-8-6 was used as a control and transformed with plasmids containing the *flhA* (*flhA*-KO) and *sctT* (TH22–6) genes. The mutations in the *flhA* gene (*flhA*-KO) and *sctT* (TH22–6) gene were complemented by the introduction of the *flhA* and *sctT* genes, and the effects of these respective genes on bacteriocin production were evaluated.

**Fig. 4:**
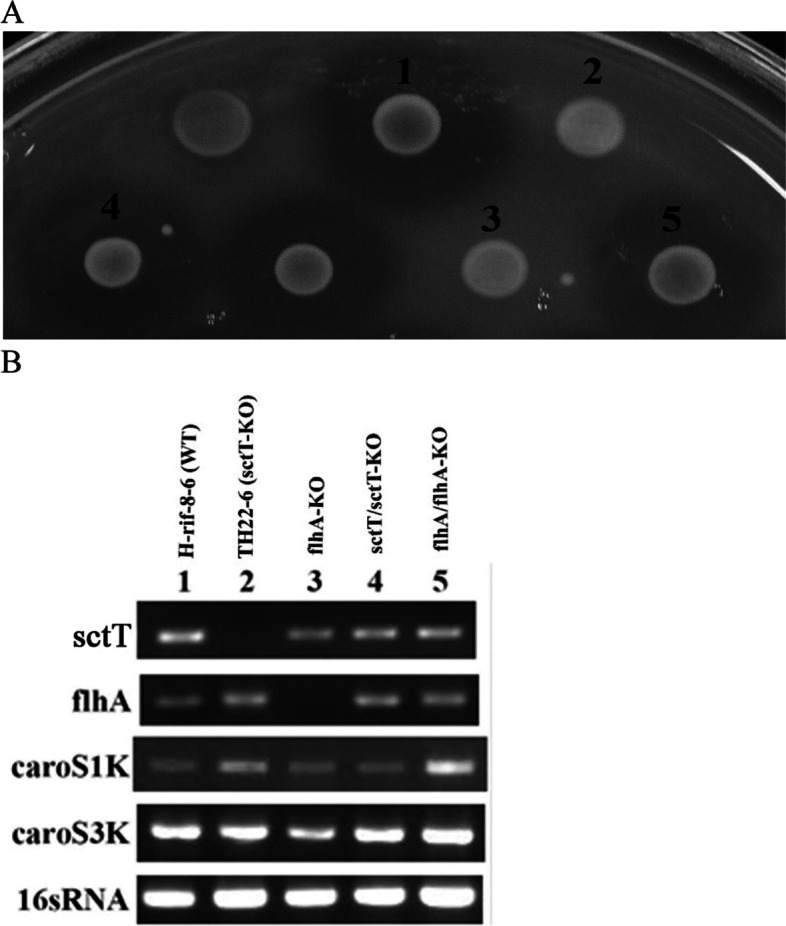
Bacteriocin assay and transcription analysis for *sctT*, *flhA, caroS1K* and *caroS3K*. **A** Bacteriocin Assay for complementation of *sctT* and *flhA:* 1, H-rif-8-6 (wild type); 2, TH22–6 (*sctT*-KO); 3, *flhA*-KO; 4, *sctT/sctT*-KO; 5, *flhA/flhA*-KO. **B** Transcription analysis of the *sctT*, *flhA*, *caroS1K* and *caroS3K*

Neither the TH22–6 (*sctT*-KO, *sctT*::Tn5) nor *flhA*-KO (*flhA*::Kan) mutant strains could secrete bacteriocins Carocin S1 and Carocin S3. However, the ability of these strains to secrete bacteriocins was recovered after complementation by transformation with the *flhA* and *sctT* genes, respectively, which have shown inhibition zone diameters comparable in size to that of wild type (Fig. 4A). After transformation, all deletion strains harboring their respective complementing plasmids secreted LMWB (Fig. 4B). It can be observed that knocking down *sctT* and *flhA* disables the extracellular export of bacteriocins Carocin S1 and Carocin S3 (Fig. 4A, No. 2 and 3). However, it can be observed that the bacteriocins are still expressed as displayed in the RT-PCR experiment (Fig. 4B, No. 2 and No. 3).

### Deletion of the T3aSS and T3bSS-related genes and bacteriocin activity assay

We knocked out several genes making up the flagellar and injectisome T3SS to find out how the deletion affects the bacteriocin secretion. The results have shown that deletion of *sctU*, *sctV*, *flgG, fliE* and *fliR* genes disabled the secretion of bacteriocin. This suggests that these genes are essential and are required in the secretion of Carocin.

**Fig. 5:**
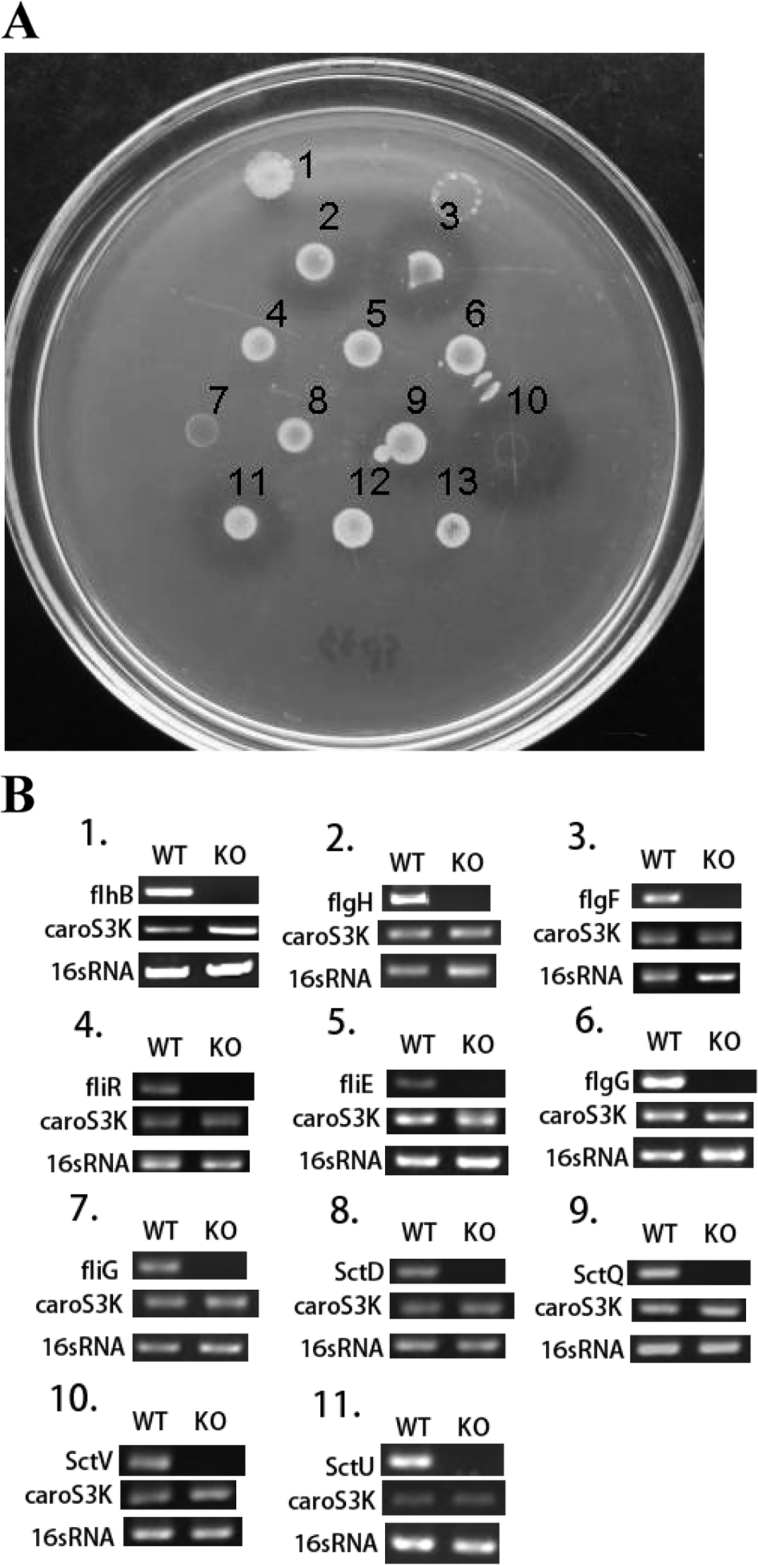
Bacteriocin assay and transcription analysis for several Type-IIIa and Type-IIIb SS related genes. **A** Bacteriocin assay and deletion of various Type-IIIa and Type-IIIb SS related genes. 1, *Serratia marcescens* (Marker); 2, H-rif-8-6 (wild type); 3, *flhB-*KO; 4, *flgH-*KO; 5, *flgF-*KO; 6, *fliR-*KO; 7, *fliE-*KO; 8, *flgG-*KO; 9, *fliG-*KO; 10, *sctD-*KO; 11, *sctQ*-KO; 12, *sctV-*KO; and 13, *sctU-*KO*.***B** Transcription analysis of 1, *flhB*; 2, *flgH*; 3, *flgF*; 4, *fliR*; 5, *fliE*; 6, *flgG;* 7, *fliG*; 8, *sctD;* 9, *sctQ*; 10, *sctV*; and 11, *sctU*

Results from our previous research have shown the significance of *flhA*, *flhC*, *flhD,* and *fliC* genes in regulating the synthesis of bacterial flagella in *Pcc* and that Carocin S1 utilizes this secretion machinery (T3bSS) [20]. The results of the present study have shown that further deletion of T3bSS-related genes such as *flgG, fliE, fliR,* and *flhA* similarly disrupts the extracellular export of Carocin S1 and Carocin S3 (Fig. 5). This result further supports the previous findings that the T3bSS is used in the secretion of *Pcc* bacteriocin. However, it can also be observed that deletion of various T3aSS-related genes like the *sctT, sctU,* and *sctV* likewise affects the Carocin S1 and Carocin S3 secretion. Interestingly, deletion of the T3bSS gene *flhB* did not restrain bacteriocin secretion to the outside of the cell but knocking out its homologous gene in the T3aSS, *sctU,* inhibited the expression of the bacteriocins to the extracellular environment.

The current findings of this study have shown an interesting interaction between the T3aSS and T3bSS. From these findings, we hypothesize that the genes *sctT, sctU,* and *sctV* play important roles in the synthesis of chaperones for the delivery of bacteriocins Carocin S1 and Carocin S3.

## Discussion

This experiment uses Transposon Tn5 to disrupt the genes related to LMWB production, and TAIL-PCR method was used to amplify the PCR product (2249 bp DNA fragment) extending from both ends of Tn5. The 2249 bp DNA was found after using the Basic Local Alignment Search Tool (BLAST) by National Center for Biotechnology Information (NCBI). The part disrupted by Transposon Tn5 has a complete gene (*sctT*). The amino acid sequence of the disrupted gene is 45% identical with the YsaT protein of *Yersinia enterocolitica* and 61% identical with YsaT protein of *Sodalis glossinidius*. Literatures [14, 41] indicate that these proteins are known to be involved in the construction of T3SS. When the *sctT* gene was disrupted, *Pcc* LMWBs were no longer secreted, but HMWBs still exist as indicated by the thin inhibition zone produced around the tested strain (Fig. 1, No. 4). Hence, we speculate that the gene disrupted by Tn5 is likely to be a protein component involved in the secretion of LMWBs.

We compared the DNA fragment obtained from the chromosomal DNA of H-rif-8-6 (3031 bp DNA fragment), NCBI GenBank protein sequence alignment, and TAIL-PCR product (2249 bp DNA fragment), and the results were roughly the same. The *sctT* gene is located at the end of the 3031 bp DNA fragment. There are about two to three ORFs in the front and rear regions of the *sctT* gene. Most of these ORFs are related to the constituent proteins of the T3SS.

Much of our understanding of the T3SS is derived from the study of pathogenic bacteria and their interactions with plant and animal hosts [42]. T3SSs of bacteria are specific export machineries for virulence that allow their translocation to eukaryotic cells. The SctT protein is one of the injectisome T3SS apparatus. It is part of the inner membrane machinery of the T3aSS [43]. It belongs to the five highly conserved inner membrane proteins (SctRSTUV) which are necessary for the function of the pathogenic T3SS, though, their individual functions are unclear. It is worth noting that these injectisome proteins show a high degree of sequence homology to components of the evolutionarily related flagellar secretion system (T3bSS) and may represent a functional core, serving critical chemical roles in initiating or powering protein secretion. SctRSTUV are important in the organized, stepwise assembly of the T3aSS basal body.

The role of SctT is not well understood, but it has been identified as a T3aSS protein based on sequence homology among multiple bacterial species [44]. This study would be the first to report the function of SctT in the secretion of an effector protein. By the complementary experiment, the result showed the *sctT* gene expression was a requirement for the LMWBs, Carocin S1 and Carocin S3, secretion. The current result on the role of *sctT*, a gene that encodes a protein in the T3aSS assembly, have led us to further investigate some possible interactions between the T3aSS and T3bSS genes in the secretion of *Pcc* bacteriocins.

Previous findings [20] have shown that Carocin S1 is secreted via T3bSS. The results revealed that bacteriocin secretion and motility of the bacteria is affected by T3bSS gene knockouts. This present study, however, has shown that bacteriocin secretion is also affected by deletion of a T3aSS-related gene. We performed a motility test on the *sctT*-knockout and wildtype strains. The results have shown no significant difference on the motility of the two strains (Supplementary Fig. 4). The findings reveal that the *sctT* knockout does not interact with the flagellar function. This indicates that SctT, a T3aSS protein, interacts with the T3bSS function (secretion) but not on flagellar function (motility). According to Sun et al. [36] and Denise et al. [37], some bacteria encode multiple T3SSs (e. g. *Burkholderia*) to interact with multiple types of eukaryotic hosts. Also, according to Matsuda et al. [45], some effectors can evolve to be recognized by multiple systems (e.g. a toxin from *Vibrio)*. In this case, it might be possible that *Pcc* can also encode multiple secretion systems, and the bacteriocins Carocin S1 and Carocin S3 are recognized by both secretion machineries.

To test this assumption, we performed series of deletions of multiple genes related and significant to both the T3aSS and T3bSS. We found that deletion of T3aSS-related genes (*sctT, sctU* and *sctV*) and T3bSS-related genes (*flgG, fliE, fliR* and *flhA*) did impede the secretion of bacteriocin. Interestingly, deletion of any of the T3bSS-related gene (*flgG, fliE, fliR* and *flhA*) totally halts the secretion of bacteriocin. Similarly, omission of T3aSS-related genes (*sctT, sctU,* and *sctV*) impedes the secretion of bacteriocin. These results did not support our initial assumption. If *Pcc* is capable of encoding both types of T3SS like what Sun et al. [36] and Denise et al. [37] explained about *Burkholderia*, disabling one secretion system (*e. g.* T3aSS) could possibly not disrupt the delivery of Carocin outside the cell because the presence of the other machinery (T3bSS) serves as an alternative system for secretion. The results of the experiment, and that of the previous study [20], nevertheless, reveals that the *Pcc* bacteriocin could be secreted by a single secretion machinery that utilizes the genes related to both T3aSS and T3bSS. Thus, this led to the rejection of the assumption that *Pcc* can encode both types of T3SS.

**Fig. 6:**
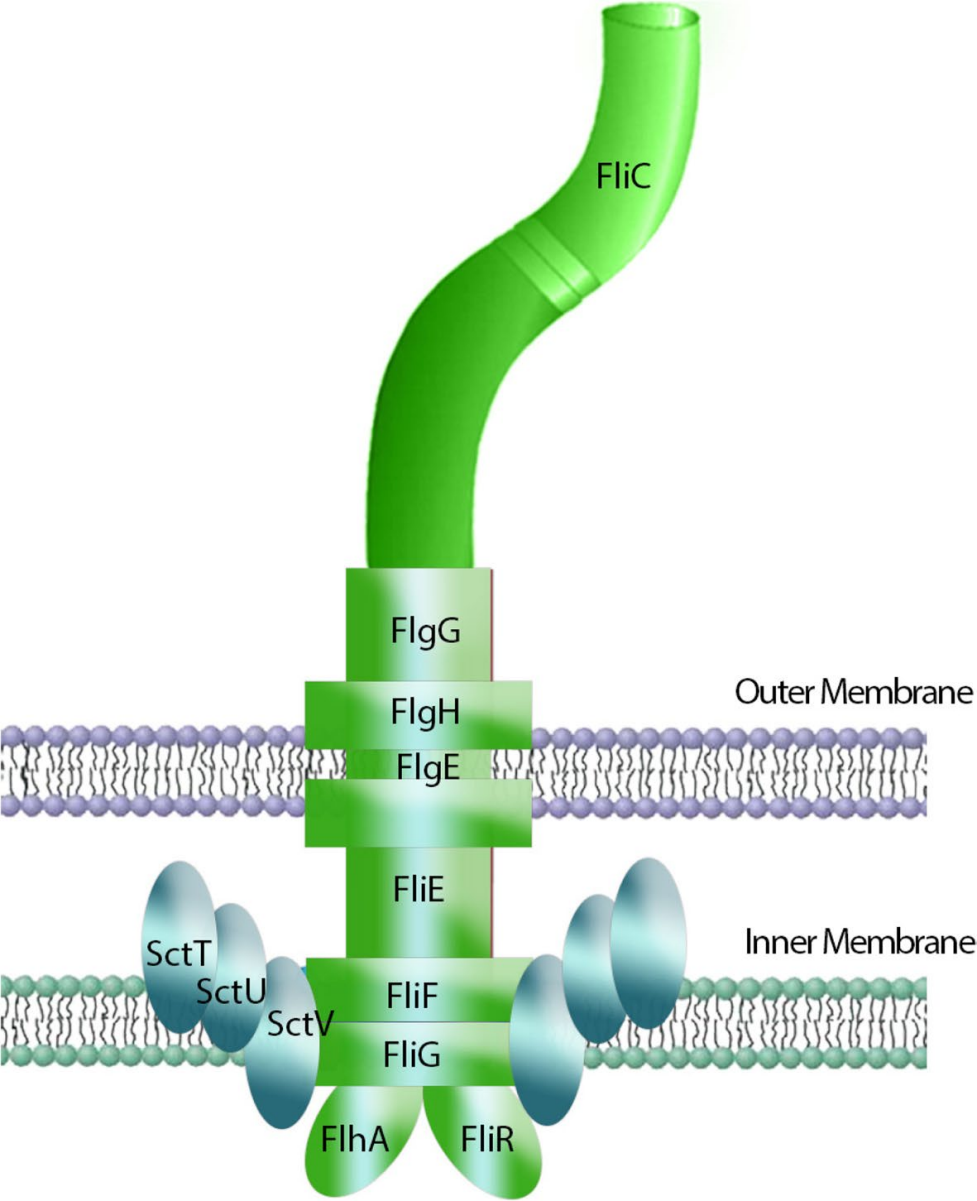
The hypothesized flagellar T3bSS with the injectisome T3SS subunits as chaperones

We therefore hypothesized that the inability of *Pcc* to secrete Carocin S1 and Carocin S3 resulted from the absence of T3aSS genes *sctT*, *sctU* and *sctV,* which encode chaperone proteins for the bacteriocin secretion via T3bSS. Figure 6 shows the hypothesized flagellar secretion system, with T3aSS proteins acting as chaperones.

This study would be the first to report the potential role of SctT, SctU and SctV T3aSS proteins in the delivery of an effector protein via T3bSS. As inferred from the results of this study, these proteins serve as potential chaperones in the secretion of bacteriocins Carocin S1 and Carocin S3 in *Pcc*. Some literatures stipulate that secretion of most effectors was reduced but not abolished in the absence of their chaperones [46–51]. Recent reviews on flagellar chaperones, however, have stated that flagellar chaperones might be required mainly to prevent premature interactions between subunits of the flagellum by masking their interaction domains [52]. The result of the present study shows that the absence of the potential chaperone genes halts the secretion of the effector proteins in *Pcc*.

The flagellar system uses cytoplasmic chaperones [53] such as the FlgN for FlgL and FlgK (hook-related proteins), FliT for FlgL and FlgK (filament-capping proteins), and FliS for FliC (flagellin protein) [54–56]. It was proposed that flagellar chaperones mediate interactions between the various subunits of the flagellum by binding to the carboxy-terminal region of the substrate [54–57]. These C-terminal domains are thought to be the glue by which individual filament subunits polymerize together during assembly of a growing flagellum [58]. Unless these domains are stabilized by a bound chaperone, the monomeric forms are swiftly oligomerized. This has been proven experimentally. Flagellar chaperones such as the FliS [55–57] and FlgN [58] prevent premature polymerization. Parsot et al. [59] explains that premature associations of flagellar proteins in the cytoplasm result in their instability.

In our experiment, deletion of genes associated with the chaperone proteins have prevented the extracellular secretion of Carocin S1 and Carocin S3. The bacteriocins have been produced but not extracellularly secreted perhaps due to non-assembly of the flagellar components essential for their secretion which could have resulted from the possible instability and premature associations of the various flagellar proteins. In this report, Carocin S1 and Carocin S3 are detected in the RT-PCR in small amounts (see Fig. 5). However, the bacteriocin assay revealed that these bacteriocins were not extracellularly secreted. Similarly, previous reports [55, 58] have shown that substrates were detected in lower amounts and were not secreted in the absence of their chaperones.

The roles of the T3aSS-related proteins, SctT, SctU and SctV, in the secretion of *Pcc* bacteriocins could be any of the common roles of chaperones in the flagellar T3SS. Whether they act to protect substrates from premature associations with self or other proteins, to guide substrates to the correct T3SS, to establish a secretion order among the substrates, or to couple gene transcription to the process of apparatus assembly, their specific function in the secretion of Carocin S1 and Carocin S3 remains an unanswered question and needs further investigation and confirmation. Hence, it is highly recommended that further studies be conducted.

These include further confirmatory tests on other T3aSS and T3bSS related genes which were not included in the study. Moreover, CoIP (Co-Immunoprecipitation) technique can also be conducted to confirm that SctTUV associate with the T3bSS, and whether a new secretion system for bacteriocin and other toxin proteins exists.

## Conclusion

As shown herein, the extracellular export of *Pcc* LMWBs Carocin S1 and Carocin S3 are dependent on secretion machinery involving genes related to both the injectisome (T3aSS) and flagellar (T3bSS) secretion systems. This study is the first to report such unique interaction between genes and proteins of both types of T3SS. Results of gene deletions revealed that *Pcc* bacteriocins are possibly secreted by the T3bSS which utilizes T3aSS-related proteins SctT, SctU, and SctV as secretion chaperones. However, how they interact with the bacteriocins, and what are their specific role as flagellar T3SS chaperones need further investigations.

## Methods

### Bacterial strains, plasmids, media, and growth conditions

The bacterial strains and plasmids used in the study are shown in Table 1. Propagation of the *Pectobacterium carotovorum* subsp. *carotovorum* strains was done in 1.4% nutrient agar (NA) at 28 °C or in LB medium with 5 g/L of NaCl with shaking. On the other hand, *E. coli* strains were propagated at 37 °C in a modified LB medium with shaking. Rifampicin, kanamycin, and ampicillin (50 mg/L) were added to the media when required.

**Table 1:** Bacteria and plasmids used in this study

Bacterium or plasmid	Relevant characteristics	Source
*E.coli*		
1830	*pro*^−^*met*^−^*Km*^r^*Nm*^r^, containing transposon	Gantotti et al. [40]
	Tn5 on the “sucidal” plasmid pJB4JI	
DH5α	*supE44hsdR17recA1endA1gyrA1thi-1relA1*	Hanahan; Reusch et al. [60, 61]
*Pectobacterium carotovorum* subsp. *carotovorum*		
89-H-4	putative biocontrol agent	Laboratory stock
H-rif-8-6	89-H-4, *Rif*^r^	this work
Ea1068	wild type	Laboratory stock
SP33	wild type	Laboratory stock
TH22–6	H-rif-8-6, *sctT::*Tn*5, Rif*^r^, *Kan*^r^	this work
FlhA-KO	H-rif-8-6, *flhA::Kan, Rif*^r^, *Kan*^r^	Chan et al. [9]
SctV-KO	H-rif-8-6, *sctV::Kan, Rif*^*r*^*, Kan*^*r*^	this work
SctU-KO	H-rif-8-6, *sctU::Kan, Rif*^*r*^*, Kan*^*r*^	this work
SctD-KO	H-rif-8-6, *sctD::Kan, Rif*^*r*^*, Kan*^*r*^	this work
SctQ-KO	H-rif-8-6, *sctQ::Kan, Rif*^*r*^*, Kan*^*r*^	this work
FlgH-KO	H-rif-8-6, *flgH::Kan, Rif*^*r*^*, Kan*^*r*^	this work
FlgF-KO	H-rif-8-6, *flgF::Kan, Rif*^*r*^*, Kan*^*r*^	this work
FlhB-KO	H-rif-8-6, *flhB::Kan, Rif*^*r*^*, Kan*^*r*^	this work
FliR-KO	H-rif-8-6, *fliR::Kan, Rif*^*r*^*, Kan*^*r*^	this work
FliG-KO	H-rif-8-6, *fliG::Kan, Rif*^*r*^*, Kan*^*r*^	this work
FliE-KO	H-rif-8-6, *fliE::Kan, Rif*^*r*^*, Kan*^*r*^	this work
FlgG-KO	H-rif-8-6, *flgG::Kan, Rif*^*r*^*, Kan*^*r*^	this work
Plasmid		
pACYC177	*Amp*^r^,*Kan*^r^, low copy number	Chang et al. [62]
pBR322	*Amp*^r^,*Kan*^r^	Bolivar et al. [38]
pB47	*Amp*^r^,*Kan*^r^, 3.0 kb DNA fragment contain *sctT*	this work
pBSCTT	*Amp*^r^, *sctT*	this work
pBFA	*Amp*^r^, *flhA*	this work

*Amp*^*r*^ indicates ampcillin resistance, *Rif*^*r*^ indicates rifampicin resistance, and *Kan*^*r*^ indicates Kanamycin resistance

### Bacterial mating

For bacterial mating, the membrane-filter method was carried out as described by Gantotti et al. [40]. Overnight cultures of *Pcc* (recipient) H-rif-8-6 and *E. coli* (donor) 1830 were evenly spread onto 0.22 μm pore size membrane filters (Millipore, Inc. Bedford, MA) which were placed on LB agar media and incubated overnight at 28 °C. After conjugation, suspensions of the progeny were appropriately diluted and were grown at 28 °C for 24 to 48 h on modified Drigalski’s agar plates (with rifampicin and kanamycin, 100 μg ml-1). Colonies were isolated for the bacteriocin production test.

### Bacteriocin assays

Bacteriocin production of the isolates was examined using the double-layer method as previously described [38, 39]. The isolates were placed on hard IFO-802 (with 1.4% agar) and soft IFO-802 (with 0.65% agar) medium. The cells were first incubated for 12 h for colonies to form. The colonies were then exposed to ultraviolet irradiation before incubating for another 12 h. Thereafter, the cells were treated with chloroform and finally covered with soft agar containing the indicator cells. An inhibition zone of indicator-cell (SP33 or Ea1068) growth around the colony indicates bacteriocin production.

### Preparation of genomic DNA, plasmid DNA and RNA

The procedures of plasmid preparation, genomic DNA isolation, and DNA manipulation were performed according to Sambrook [63]. For RNA preparation, exponentially growing *E. coli* DH5α cells were harvested (OD_595_ of about 6.0). Trizol reagent (Invitrogen, USA) was used in isolating the RNA which was resuspended in diethylpyrocarbonate (DEPC)-treated water. RNA concentration was determined at OD_260_ absorption and was analyzed by electrophoresis on 1.5% formaldehyde-morpholinepropanesulfonic-agarose gel.

### TAIL-PCR and restriction DNA library screening

Previously detailed protocols were utilized for the general polymerase chain reaction (PCR) [63] and thermal asymmetric interlaced PCR (TAIL-PCR) [64].

Specific primers at both ends of Tn5, namely PF1, PF2, PF3, PR1, PR2, and PR3 were designed for TAIL-PCR. Using the above primers for amplification, two PCR products with unknown DNA sequences at both ends of Tn5 were obtained. However, after the sequence analysis, it was found that the two segments were the same PCR products. Hence, two sets of two PCR products were designed using this known DNA sequence of about 300 bp.

ABI PRISM Dye Terminator Cycle Sequencing Ready Reaction kit (Applied Biosystems, Foster City, CA) was used in the sequence analysis of the TAIL-PCR products. Cycle sequencing was carried out in a GeneAmp System 9600 thermocycler (Applied Biosystems). The sequencing was carried out using an ABI 373S automated DNA sequencer 373S (Applied Biosystems) based on the manufacturer’s protocol.

### Hybridization and Southern blots

Southern blots were performed according to the DIG Application Manual (Roche, USA). A 543-bp DNA fragment (431 probe) was amplified with pJI and pBI primers, subcloned into pGEM-T Easy vector (Promega Inc., USA), and labelled using a Random Primed DNA Labeling Kit (Roche Diagnostics, USA). The genomic DNA of the wild-type strain H-rif-8-6 was digested with various restriction endonucleases, with sites located outside the putative open reading frame. Samples were electrophoresed and analyzed with Southern blotting. After detection using the 431 probe, the DNA from positive gel slices was purified and cloned into pBR322 to give the carocin-producing plasmid pB47. The pB47 construct was isolated and detected as above with the 431 probe.

Southern and colony hybridizations, probe labeling, and detection were performed by using a DIG DNA Labeling and Detection kit (Boehringer Mannheim GmbH, Mannheim, Germany). Hybridization was performed overnight, and the membrane was washed according to the recommendations of the manufacturer. DNA electrophoresis, restriction digestion, ligation, and transformation for *E. coli* were carried out as described by Sambrook et al. [63]. Plasmid DNA transformation for *Pcc* was performed by the methods of Hinton et al. [65] and Hanahan [60].

### Subcloning of *sctT* gene from H-rif-8-6

The DNA fragment of *sctT* was amplified by PCR from H-rif-8-6 using oligonucleotide primers SctT-sen and SctT-anti*.* The PCR product was subcloned into pGEM-T Easy vector by TA cloning (Promega Inc., USA). The *sctT* gene containing product was digested with restriction enzymes *Hind*III and subcloned into plasmid pBR322. The new plasmid was designated pBSctT. One hundred transformed colonies were isolated using selective LB agar containing 100 μg/ml of ampicillin after the transfer of pBSctT into *E. coli* DH5α. The presence of the *sctT* gene was detected by colony hybridization using the 431 probe and electrophoresis after digestion with *Hind*III to yield the expected 10-Kb DNA fragment bearing SctT protein. The pSctT plasmid was isolated from DH5α/pSctT and transferred into the insertion mutants of *Pcc* TH22–6. One hundred colonies were isolated by selection on modified Drigalski’s medium containing 50 μg/ml of kanamycin, rifampicin, and ampicillin. The *sctT* gene was detected as previously described.

### Construction of the null alleles of *flhB*, *flgH*, *flgF*, *fliR*, *fliE*, *flgG*, *fliG*, *sctD*, *sctQ*, *sctV*, and *sctU* genes

Primers were designed based on the gene sequences available in the database (*E. coli, P. aeruginosa*, *Shigella* and *Yersinia*). Thereafter, using the genomic DNA of *Pcc*, we performed PCR to amplify the target genes and the gene sequences were confirmed. After the confirmation, the genes were introduced to the vector pBR322, then pBflhB, pBflgH, pBflgF, pBfliR, pBfliE, pBflgG, pBfliG, pBsctD, pBsctQ, pBsctV, and pBsctU constructs were obtained.

The various genes were isolated from these constructs by digesting with different restriction enzymes which cleave at two sites in the constructs and thereby conveniently delete the target genes from the operon. The resulting plasmid were designated and labelled accordingly.

A kanamycin-resistant gene from pACYC177 was isolated, made blunt-ended using a DNA-blunting kit (Takara Co., Tokyo, Japan), and inserted in the unique restriction site of the target genes. The resulting plasmid was designated consequently (see Table 1). The gene-Kan was re-isolated and linearized after restriction enzyme digestion, which deleted the ampicillin resistance gene and replication site of the plasmid.

The linearized construct was transferred into H-rif-8-6, resulting in the homologous replacement of the native genes and generating null alleles. DNA fragments were introduced into *Pcc* strains using electroporation (1.25 kV/cm, 200 Ω, 25 μF) according to Metzger et al. [66]. Plasmid DNA transformation for *Pcc* was performed using the previously described method by Hinton et al. [65] following an incubation at 35 °C until the optical density (550 nm) of the culture was 0.40 to 0.55.

The DNA fragments of the target gene were amplified by PCR from Hrif-8-6. After PCR amplification using pairs of oligonucleotide primers (see Table 2), the partial DNA fragments were isolated and subcloned into plasmid pBR322 to generate the needed plasmid.

**Table 2:** Primers used in this study

Primer^a^	Sequence (5′ → 3′)
PR-1	5′- GCCGAAGAGAACACAGATTTAGCCCA
PR-2	5′- CCGCACGATGAAGAGCAGAAGTT
PR-3	5′- CAGATCTCTGGAAAACGGGAAAGG
PF-1	5′- AGAGAACACAGATTTAGCCCAGTCGG
PF-2	5′- CCGCACGATGAAGAGCAGAAGTTAT
PF-3	5′- GATCCTGGAAAACGGGAAAGGTTC
N-1	5′- NGTCGA(G/C)(A/T)GANA(A/T)GAA
N-2	5′- GTNCGA(C/G)(A/T)CANA(A/T)GTT
N-3	5′- (A/T)GTGNAG(A/T)ANCANAGA
P-3	5′- CTCGACGTTGTCACTGAAGCGGGAAG
P-4	5′- AAAGCACGAGGAAGCGGTCAGCCCAT
PJ1	5′-GTTTTTTCAGCCATTGTCGC
PB1	5′-TCTGGCTTTCTGAACTTTGC
sctT-sen	5′-TGAAGCTTATGAGCCCAGTG
sctT-anti	5′-TAATAAGCTTTGGTGCAGCC
flhA-sen	5′-TCACTCAACGTTGCATCTAC
flhA-anti	5′-CAAGATGTTGGCCAACAGATG
sctV-sen	5′-TCCGATATAGGTGTTGAGGC
sctV-anti	5′-CCAGCCAGTTAATGATGTGC
sctU-sen	5′-CAATCGTCCTGAACTGTTGG
sctU-anti	5′-GTACTGACtGCACCATGCTC
sctD-sen	5′-GAAGCACCGTGGTGTTGAAG
sctD-anti	5′-GCCTTCTGGATAGCGTTGATG
sctQ-sen	5′-CGTGGCAGCCACTCGAGTGTGAAC
sctQ-anti	5′-GCAGACAAGGATCCTCAGCGGAATC
flgH-sen	5′-ATGGCGAATAAATGGCGTTG
flgH-anti	5′-GGAAGAAACGCTGTAACCAC
flgF-sen	5′-GAATCGCAGCCTGTCACAAC
flgF-anti	5′-TGTGCCAGATCCTCTGCAAG
flhB-sen	5′-AAACAGAAGCTTCCACTCCC
flhB-anti	5′-ACGCCGCAGTCGACGCTTC
fliR-sen	5′-TGATACCAGCCAACTCAGTC
fliR-anti	5′-CCAGCCGATCAAAGAATTCG
fliG-sen	5′-ATGACCCTGACAGGAACAG
fliG-anti	5′-TTAGACATAAGCATCCTCGC
fliE-sen	5′-CAGTCGCTATGTCCCCTAAC
fliE-anti	5′-TACACCTGCATGCTCATCAC
flgG-sen	5′-TACCCGTGACGGTTCATTTC
flgG-anti	5′-TTGTAGCATCTGATCATACG

^a^All primers were purchased from MDE Bio Inc., Taipei, Taiwan

### RNA preparation and RT-PCR

Bacteriocin synthesis medium (BSM; 0.5% sucrose, 0.1% NH_4_Cl, 0.2% KH_2_PO_4_, and 0.02% MgSO_4_·7H_2_O (pH = 7.5) was used to produce bacteriocins Carocin S1 and Carocin S3. Total RNA was extracted from cells (*Pcc* harboring constructs) that were grown without antibiotics at 28 °C. To determine the stability of the strains, culture samples (8 ml each; with rifampicin [0.2 mg/ml] added when cell density was ~ 150 Klett units to block bacterial contamination) were withdrawn at various time points and transferred to tubes containing 5 mL of ice-cold water. Total RNA was extracted using Trizol (Invitrogen, Carlsbad, CA) according to the manufacturer’s protocol.

On the other hand, the Reverse Transcription-PCR (RT-PCR) using AMV Reverse Transcriptase (Promega, USA) was carried out based on the instructions provided by the manufacturer. One microgram (1 μg) of RNA was subjected to RT-PCR. Reverse primer (see Table 2) was used in first strand cDNA synthesis. The RT mixtures were diluted and used as templates in a PCR reaction with the pairs of primers (Table 2).

### Computer analysis of sequence data

The nucleotide sequence and the deduced amino acid sequence of *sctT* were compared using the BLAST and FASTA programs of the National Center for Biotechnology Information server (National Library of Medicine, USA). Sequence data were compiled by DNASIS-Mac software (Hitachi, Tokyo, Japan).

## Supplementary Information

**Additional file 1: Supplementary Fig 1.** The Thermal Asymmetric Interlaced PCR (TAIL-PCR) [63] and the results from the process. (a) Schematic diagram of the TAIL-PCR process and the thermal conditions [63]; (b) Unknown nucleotides are amplified by three contiguous specific primers from Tn5 insert end sequence and using the same arbitrary primer per reaction; (c) Results from the TAIL-PCR. Lane 1 L1, indicate primary reaction, TH22–6 chromosome DNA as template DNA, that use specific primer PL1 and arbitrary primer N1; Lane 2 L1, indicate secondary reaction, dilute primary PCR product as template DNA, that use specific primer PL2 and arbitrary primer N1; Lane 3 L1, indicate tertiary reaction, dilute secondary PCR product as template DNA, that use specific primer PL3 and arbitrary primer N1. Another set of lanes 1A2 to 3A2 followed the same procedure and used the PA1 to PA3 and N3 as primers. All the tertiary TAIL-PCR products were sequenced with ABI sequencing system.

**Additional file 2: Supplementary Fig 2.** Southern hybridization of the wild-type strain genomic DNA. H-rif-8-6 genomic DNA was digested with a various restriction endonuclease. The enzymes used, from right to left, were 1, *EcoR*I; 2, *BamH*I; 3, *Hind*III; 4, *Pvu*II; 5, *Xba*I; 6, *Nco*I; and 7, *Not*I. We used the 1 kb marker and control (probe construction). The red arrow shows the response, and its length is about 3000 bp. The probe design was based on the sequence of TAIL-PCR near transposon Tn5. The response slice was cut from agarose gel and the DNA fragment was cloned into pBR322 vector.

**Additional file 3: Supplementary Fig 3.** Gel analysis of genome library. This was performed to screen a collection of clones for the sequence of interest, using the sequence from TAIL-PCR as a probe. All constructions were checked with endonuclease restriction enzyme *Hind*III. After electrophoresis and southern hybridization, the result showed a response construction (No. 47) with the same probe forward.

**Additional file 4: Supplementary Fig 4.** Motility Assay. The strains were tested for motility in IFO-802 medium containing 0.5% agar, incubated at 28 °C for 20 days. (A) H-rif-8-6 (parent), (B): TH12–2 (*flhC*-KO); and (C) TH22–6 (*sctT*-KO).

## Data Availability

The datasets used and analysed during the current study are available from the corresponding authors on reasonable request. The GenBank accession number of the sequence of the *sctT* gene is MZ359080.

## Abbreviations

HMWB: High-Molecular-Weight Bacteriocins
LMWB: Low-Molecular-Weight Bacteriocins
Pcc: *Pectobacterium carotovorum* subsp. c*arotovorum*
TAIL-PCR: Thermal Asymmetric Interlaced Polymerase Chain Reaction
T3SS: Type III Secretion System
T3aSS: Injectisome Type III Secretion System
T3bSS: Flagellar Type III Secretion System
