# Supplementary figures and images for: Injectisome T3SS subunits as potential chaperones in the extracellular export of Pectobacterium carotovorum subsp. carotovorum bacteriocins Carocin S1 and Carocin S3 secreted via flagellar T3SS
Source: BMC Microbiol. 2021 Dec 15;21:345. doi: 10.1186/s12866-021-02405-w (PMC8672553; doi:10.1186/s12866-021-02405-w)

**Supplementary Figure 1.**


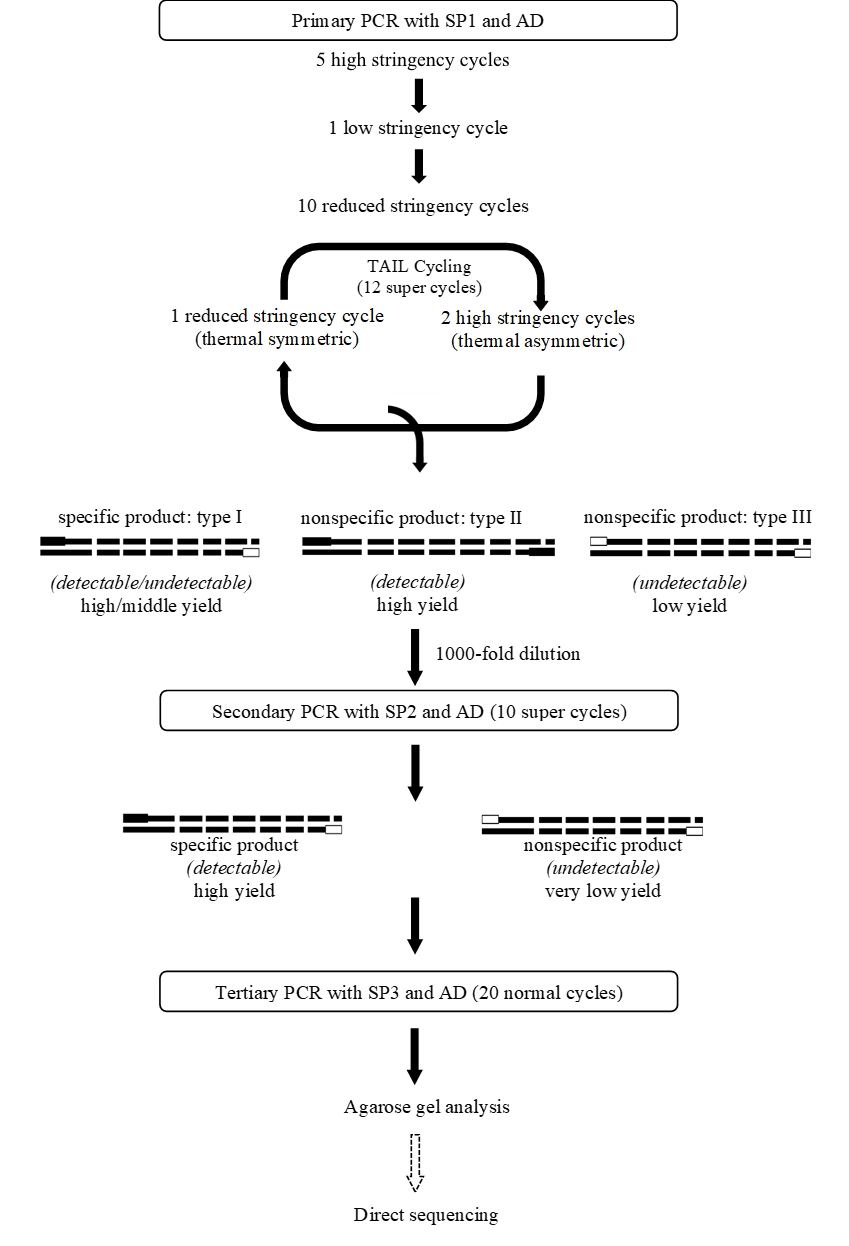


**
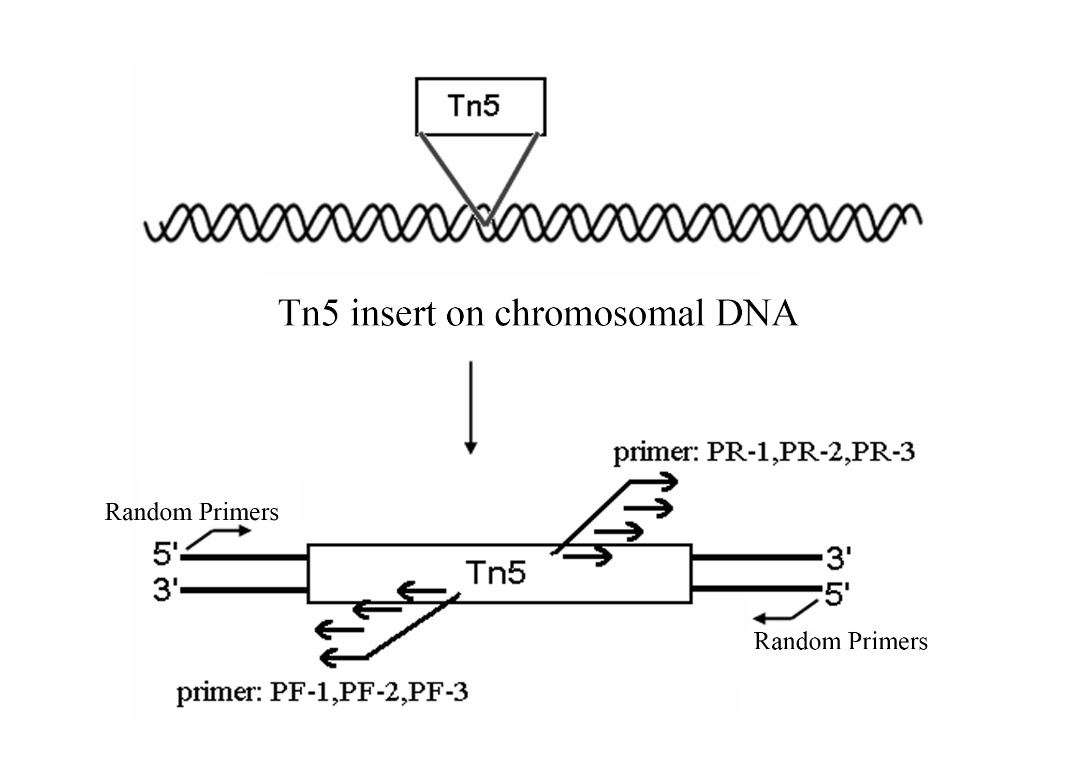
**

Supplement: Supplementary file 1 — Additional file 1: Supplementary Fig 1. The Thermal Asymmetric Interlaced PCR (TAIL-PCR) [63] and the results from the process. (a) Schematic diagram of the TAIL-PCR process and the thermal conditions [63]; (b) Unknown nucleotides are amplified by three contiguous specific primers from Tn5 insert end sequence and using the same arbitrary primer per reaction; (c) Results from the TAIL-PCR. Lane 1 L1, indicate primary reaction, TH22–6 chromosome DNA as template DNA, that use specific primer PL1 and arbitrary primer N1; Lane 2 L1, indicate secondary reaction, dilute primary PCR product as template DNA, that use specific primer PL2 and arbitrary primer N1; Lane 3 L1, indicate tertiary reaction, dilute secondary PCR product as template DNA, that use specific primer PL3 and arbitrary primer N1. Another set of lanes 1A2 to 3A2 followed the same procedure and used the PA1 to PA3 and N3 as primers. All the tertiary TAIL-PCR products were sequenced with ABI sequencing system. [file 12866_2021_2405_MOESM1_ESM.docx]

**
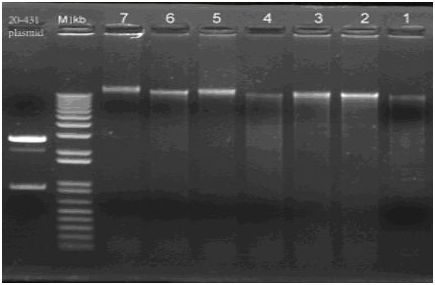
Supplementary Figure 2**


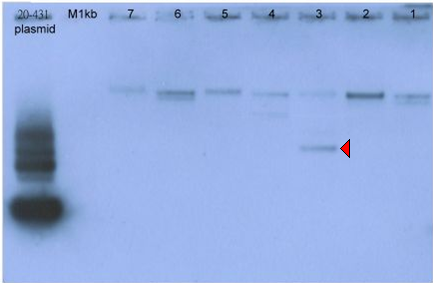

Supplement: Supplementary file 2 — Additional file 2: Supplementary Fig 2. Southern hybridization of the wild-type strain genomic DNA. H-rif-8-6 genomic DNA was digested with a various restriction endonuclease. The enzymes used, from right to left, were 1, EcoRI; 2, BamHI; 3, HindIII; 4, PvuII; 5, XbaI; 6, NcoI; and 7, NotI. We used the 1 kb marker and control (probe construction). The red arrow shows the response, and its length is about 3000 bp. The probe design was based on the sequence of TAIL-PCR near transposon Tn5. The response slice was cut from agarose gel and the DNA fragment was cloned into pBR322 vector. [file 12866_2021_2405_MOESM2_ESM.docx]

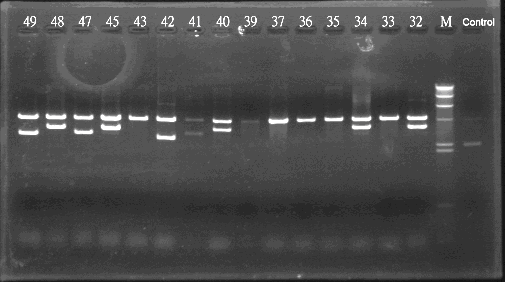
SUPPLEMENTARY FIGURE 3


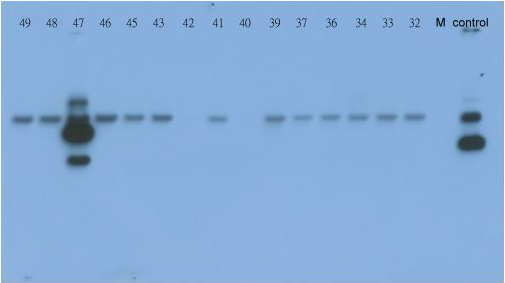

Supplement: Supplementary file 3 — Additional file 3: Supplementary Fig 3. Gel analysis of genome library. This was performed to screen a collection of clones for the sequence of interest, using the sequence from TAIL-PCR as a probe. All constructions were checked with endonuclease restriction enzyme HindIII. After electrophoresis and southern hybridization, the result showed a response construction (No. 47) with the same probe forward. [file 12866_2021_2405_MOESM3_ESM.docx]

Supplementary Figure 4


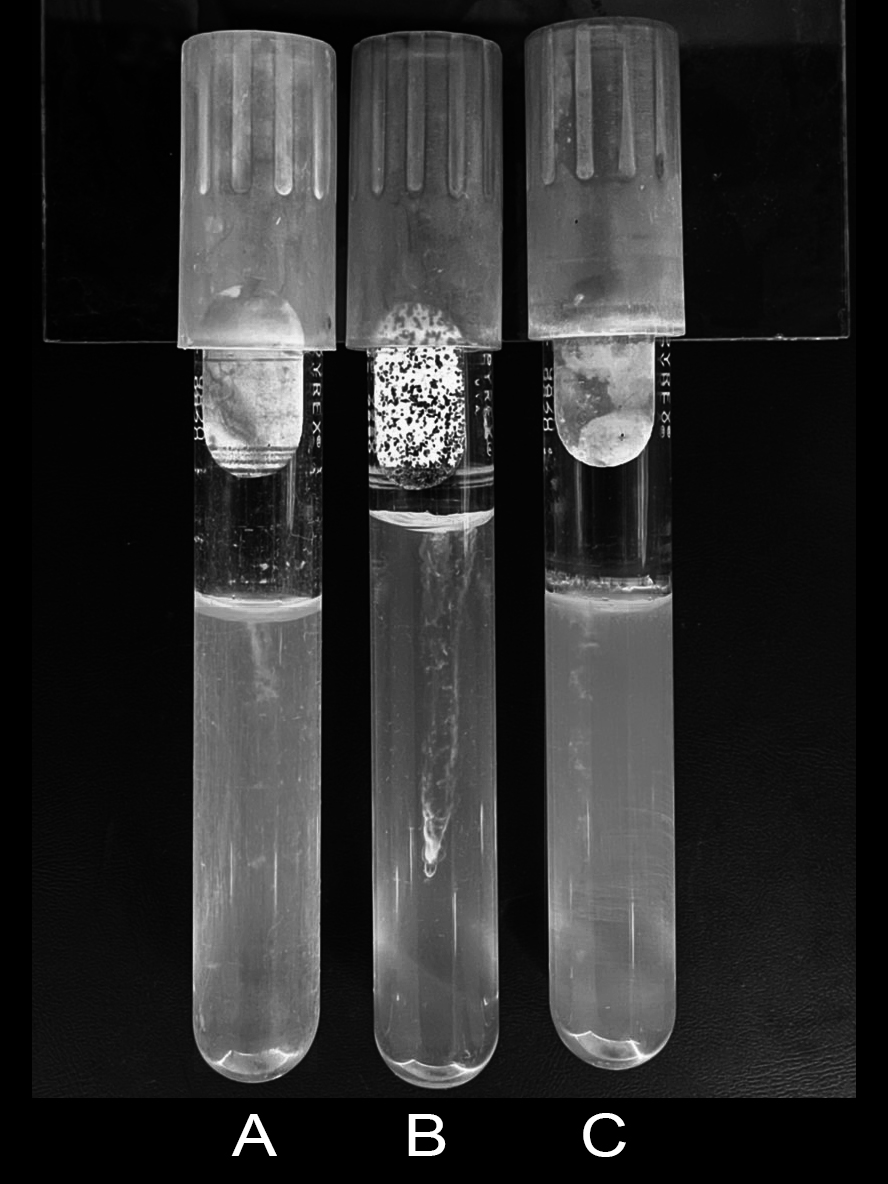

Supplement: Supplementary file 4 — Additional file 4: Supplementary Fig 4. Motility Assay. The strains were tested for motility in IFO-802 medium containing 0.5% agar, incubated at 28 °C for 20 days. (A) H-rif-8-6 (parent), (B): TH12–2 (flhC-KO); and (C) TH22–6 (sctT-KO). [file 12866_2021_2405_MOESM4_ESM.docx]
